# Cultural Differences in Visual Contents in Picture Books

**DOI:** 10.3389/fpsyg.2020.00304

**Published:** 2020-02-25

**Authors:** Megumi Kuwabara, Jannette Alonso, Darlene Ayala

**Affiliations:** California State University, Dominguez Hills, Carson, CA, United States

**Keywords:** cross-cultural, visual environment, children, picture books, infant/toddler, preschool

## Abstract

Previous studies investigating cultural differences in attention and perception have shown that individuals from Western countries (e. g., the U.S.) perceive more analytically whereas individuals from East Asian countries (e.g., Japan) perceive more holistically (e.g., Nisbett and Miyamoto, 2005). These differences have been shown in children as young as 3 years old (Kuwabara and Smith, 2016). To reflect cultural influences on cognition, specifically on attention and perception, this study investigated potential differences in the visual environment. In this study, we focused on one of such visual environments that young children are exposed to regularly and influence other domains of development, picture books (Horst and Houston-Price, 2015). Thirty seven U.S. picture books and 37 Japanese picture books were coded for visual contents—how visually crowded—by computer software from the National Institute of Health (NIH) and human coders. Results show that the U.S. picture books are more visually crowded than the Japanese books by the software, but contained more objects than the Japanese books as expected, which reflect well with the cultural differences in attention observed in young children in previous studies. However, the results differed based on the target ages of the books. The implication of the current study is discussed as a reflection of the mutual constitution between culture and psyche.

Cultural influences can be observed in how our minds work and process information and how the differences in the environment might trigger or facilitate those differences in our minds (Markus and Hamedani, 2007). Both of these processes in mind and environment must be studied systematically if we would like to untangle the cultural influences on human cognition. Cultural differences in our cognition, specifically in attention and perception have been documented in a wide variety of tasks, such as visual recognition tasks (e.g., Masuda and Nisbett, 2001; Kitayama et al., 2003), including eye-tracking method (e.g., Chua et al., 2005; Kelly et al., 2010) and brain imaging (e.g., Hedden et al., 2008; Masuda et al., 2014). Accumulating evidence suggests individuals in Western cultures tend to focus on individual and most salient elements of a scene (analytic attention) while individuals in East Asian cultures tend to focus on the relationship among objects in a scene (holistic attention) (see Nisbett and Miyamoto, 2005 for review). For example, when shown a mundane scene of fish in an aquarium, adults from Western cultures were more likely to look at and remember the focal object, whereas adults from East Asian cultures were more likely to fixate on and remember multiple elements in the scene and their relation to each other (Masuda and Nisbett, 2001). Although the number of cross-cultural studies on attentional differences in young children is yet limited, the findings that children developing in East Asian cultures are more sensitive to relations among objects in scenes than are children in Western cultures, the trend similar to adults, have been observed as young as 3 years of age (Duffy et al., 2009; Kuwabara and Smith, 2012, 2016; Moriguchi et al., 2012; Imada et al., 2013; Senzaki et al., 2014). The research investigating the cultural influences in attention has focused mainly on how our minds process information (in our minds differences). As pointed out by Morling and Lamoreaux (2008), if we truly want to understand the cultural influences on cognition, it is important to understand how environmental factors facilitate such cultural differences in our minds from such a young age.

Some studies have suggested and investigated how differences in visual environments we encounter daily might influence our attention (Miyamoto et al., 2006). For example, Miyamoto et al. (2006) found the U.S. street scenes are visually simpler (e.g., less crowded, a smaller number of objects) than Japanese street scenes (e.g., more crowded, a greater number of objects). Cultural products that we encounter daily also incorporate cultural values (Morling and Lamoreaux, 2008), such that the meta-analysis of previous studies of cultural products have shown the products from the Western societies included more independent values (e.g., uniqueness) whereas the products from the Eastern societies included more interdependent values (e.g., relationships). Cultural products reflect not only those cultural values, but also reflect the attentional differences observed in each culture, such as websites (Wang et al., 2012) and arts (Masuda et al., 2008) follow the similar trend—the U.S. products were visually less crowded than Japanese products. For example, the U.S. comic books framed scenes by focusing on individual characters whereas Japanese comic books framed scenes by highlighting the relationship between the characters and scenes (Cohn et al., 2012). For children, the textbooks used in elementary schools show a similar trend—the U.S. textbooks included a fewer number of characters than the Japanese textbooks (Imada, 2012). All of these trends suggest the visual environment that we encounter daily might encourage the cultural differences observed in attention and perception. Specifically, having less crowded visual environment which is commonly seen in the U.S. might encourage analytic attention because it might be easier to focus on focal objects in scenes whereas having more crowded visual environment which is commonly seen in Japan might encourage holistic attention, which might benefit the processing of the environment where objects are scattered around. A previous study (Miyamoto et al., 2006) has shown priming participants with the crowded visual scenes (e.g., Japanese street scenes) made participants (from both the U.S. and Japan) attend more holistically than priming participants with the less crowded visual scenes (e.g., the U.S. street scenes), suggesting the visual environment that we encounter daily might influence how we process information visually.

However, most studies investigating the cultural differences in visual environments focused on the visual environments that adults and older children (e.g., elementary school) encounter and few studies, if any, focused on the visual environment that young children encounter. Given cultural differences in attention have been observed as young as 3 years of age (Kuwabara and Smith, 2016), visual environments that might encourage these differences in such a young age should be a priority in untangling how visual environments might interact with attentional differences. Therefore, for this study, we focused on one of such visual environments that young children encounter often, picture books, to see whether cultural differences in visual contents, specifically, visual crowdedness, could be observed. We chose the picture book because the activities related to the picture book (e.g., looking at the picture book) is a very common activity that children often enjoy (Horst and Houston-Price, 2015). Research has also shown picture books help children's development of language (e.g., Bus et al., 1995), socio-emotional understanding (e.g., Adrian et al., 2005), and memory (e.g., Cornell et al., 1988). Picture books are also cultural products that represent culture and cultural values. For example, Yannicopoulou (2013) found the aesthetics and drawings in the book shows how different cultures see beauty, understand what good and bad are. The picture books have also shown to portray the cultural differences in ideal emotional states. For example, Tsai et al. (2007) have found the U.S. picture books contained more arousing emotions (e.g., excited) and activities (e.g., splashing and jumping in the pool) than the Taiwanese books did. In the current study, we analyzed the visual contents of the U.S. and Japanese picture books for young children to see whether visual contents follow a similar trend found in previous studies that might encourage the attentional differences observed in young children and adults. We focused on the visual contents of illustration, rather than the wording of picture books because young children spent most of the time on the illustration than the wording of the picture books (An et al., 2017). Based on Miyamoto et al. (2006), if the visual environment influences the differences in attention, the picture books from the U.S. would be less visually crowded than Japanese picture books. We also explored the potential differences in visual content based on the target age of books (targeting infant and toddlers and targeting preschoolers) to see how early visual content in the picture books might follow the similar trend found in previous studies to support the attentional differences observed in young children and adults. For picture books targeting preschoolers, we expected the U.S. picture books would be less visually crowded than the Japanese picture books following the similar trends observed in previous studies (e.g., Miyamoto et al., 2006) because cultural differences in attention have been observed as early as age three. Due to the lack of research with infants and toddlers on attentional differences, we did not have a specific hypothesis for picture books targeting infants and toddlers.

## Methods

### Sampling Books

**Figure 1:**
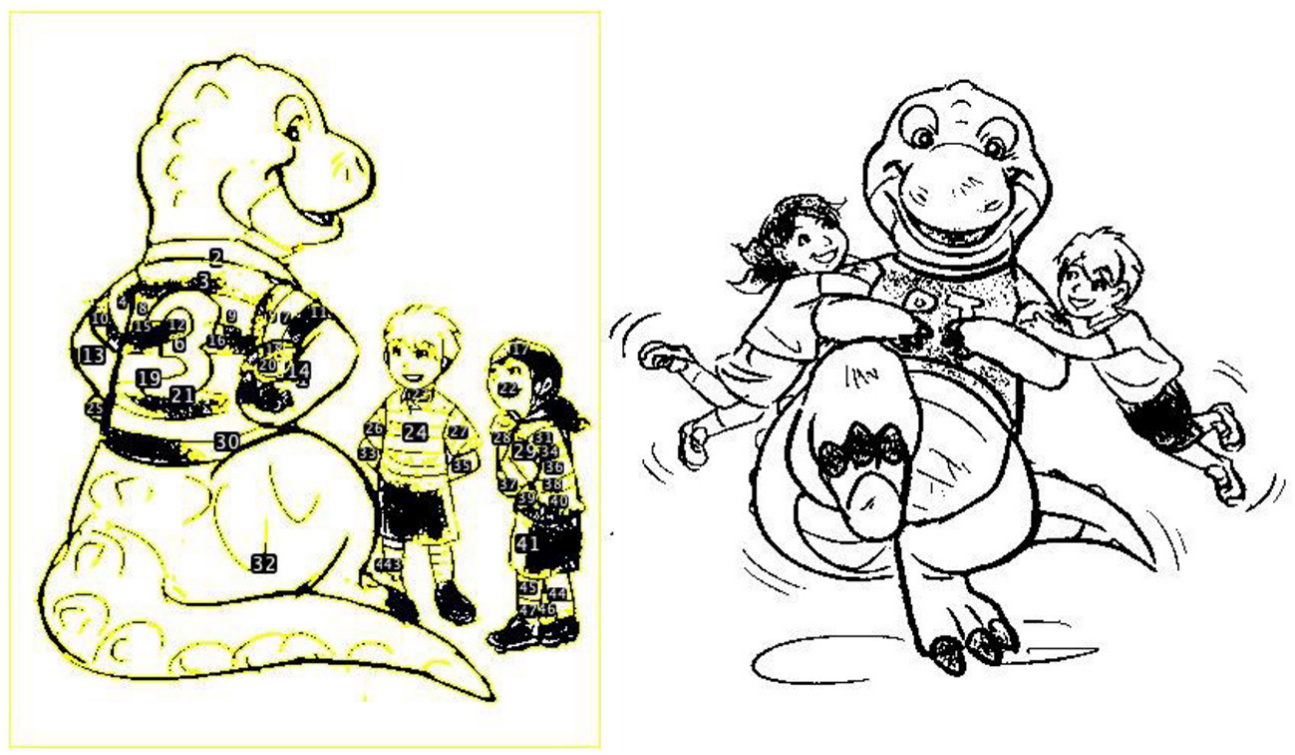
The example illustration processed by the ImageJ. The example illustration is not a part of any picture books we coded, but the illustration, “Do dinosaurs play rugby?” by Stevie Mahardhika (licensed under CC BY-NC 4.0) from the Creative Commons was used as an example due to the copyrights of picture books. The image was processed to meet the requirements of the computer program, ImageJ. The example included two image segments that would be coded separately by the ImageJ and the human coders. The yellow lines highlight the particles coded by the ImageJ with the number on each particle.

One of the challenges of cultural studies, especially dealing with media, is the selection and inclusion criteria of samples (Livingstone, 2003). Sampling books that are representative of each country using the same inclusion criteria that are not culturally biased posed a challenge for our current study. For our research purpose, we wanted to select books that are accessible and available to many children in each country using the same criteria. We used the same criteria—the list of recommended books by librarians—as an indicator that these books might be available and accessible to many children in each country. For each country, we used five libraries (the New York public library, Berkeley public library, County of Los Angeles public library, Fairfield public library, and Monroe County public library for the U.S.; Fukuoka prefectural library, Shimane prefectural library, Kyoto city library, Yokohama city library, and Ehime prefectural library for Japan) that posted the list of recommended books for children on their websites. From the list, books that were recommended by at least two library websites were included for this study. Books written and illustrated by foreign nationals were excluded from the selection due to the purpose of research. One book from Japan was excluded from coding and analysis because the book included mostly texts with very few images. Thirty six books (13 infant/toddler books and 23 preschool books) from the U.S. and 37 books (14 infant/toddler books and 23 preschool books) from Japan were included for this study (see Appendix A for the list of books). For this study, we coded each image segment on each page. The image segment was defined by the illustration on each page. For example, if the page included multiple-segmented illustrations divided by texts or the main character showing up in two separate illustrations on the page, each of those segments was counted as an image segment and coded separately from the other image segments on the page (see Figure 1 for an example). The total number of image segments coded was 780 for the U.S. books and 504 for the Japanese books. This difference is due to the number of pages books had. On average, the U.S. books had 21.17 pages (ranging between 10 and 57 pages) and the Japanese books had 16.83 pages (ranging between 11 and 26 pages). We focused on the visual contents of illustration rather than the wording of each image segment as similar to the previous study (Tsai et al., 2007) because young children spent most of the time on the illustration than the wording of the picture books (An et al., 2017). The target ages listed by the library were used for Japanese books. For the U.S. books, libraries did not agree on the target ages of books or some libraries did not list the target ages of books. Therefore, the target ages list in the scholastic website (the world's largest publisher and distributor of children's books—https://www.scholastic.com) was used for the U.S. books.

### Coding

Previous studies have used different measures to determine the visual crowdedness of images. A previous study (e.g., Miyamoto et al., 2006) has used the computer software, NIH ImageJ program (Rasband, 2018), and counted the number of particles as a measure of the visual crowdedness whereas another study (e.g., Senzaki et al., 2014) used the number of objects as a measure of the visual crowdedness. Therefore, we used both of these measures as an indicator of the visual crowdedness—one by the computer software and the other by human coders.

For the first measure, the NIH ImageJ program (Rasband, 2018) was used to measure the visual crowdedness of images on a Macintosh computer. The “analyze particles” command, which counts the number of particles in each image, was used. A particle is defined as any area of the image with a closed boundary. For example, each line of the dinosaur's shirt was counted as a particle in Figure 1. The minimum particle size was set at 100 pixels based on Miyamoto et al. (2006). Each image segment was converted to black and white with an 8-bit to meet the requirement of the program (see Figure 1 for an example image after conversion). Because we focused on the illustration, the text of each image segment was excluded from the analysis.

For the second measure, two human coders also coded 68 books out of 73 books (93%). These coders counted the number of objects in each image segment on a page. A namable object was defined as having a clear division from other objects. If the object or animal had an atypical feature (e.g., a t-shirt on the dinosaur in Figure 1) or variable numbers of features (e.g., number of windows in the house), those features were counted as separate objects. The overlapping particles (e.g., trees in a forest) without any clear boundary was counted as one object. Similar to particle counting by the ImageJ program, coders counted objects in each image segment on the page. Twelve books out of 73 total books (16%) were coded by both coders for reliability. The reliability of the coders was very high (Cronbach's alpha = 0.98). In Figure 1, the shirt that was counted as many particles by the ImageJ was counted as one object by the human coder. Therefore, the number of objects counted by human coders was much smaller than the number of particles counted by the ImageJ.

### Analysis Plan

To test whether the visual contents of the U.S. and Japanese picture books for young children follow the similar trend found in previous studies, we ran ANOVA with the number of particles counted by the ImageJ and the number of objects counted by the human coders as dependent variables and the country as the independent variable. Based on Miyamoto et al. (2006), our expected results were that picture books from the U.S. would include a smaller number of particles and a smaller number of objects than Japanese picture books.

To explore the potential differences in visual content based on the target age of books, the number of particles counted by the ImageJ and the number of objects counted by the human coders were entered as the dependent variables in 2 (country—the U.S. and Japan) × 2 (age—infant/toddler books and preschool books) ANOVA. Our expected results were preschool books from the U.S. would include a smaller number of particles and a smaller number of objects than Japanese picture books. For infant/toddler books, due to a lack of research with infants and toddlers on attentional differences, we did not have a specific hypothesis.

## Results

Particle counts per an image segment was entered as a dependent variable in ANOVA by country (the U.S. and Japan), which shows the U.S. picture books (*M* = 124.05, *SD* = 139.59) contained significantly more particles than Japanese picture books (*M* = 107.06, *SD* = 135.06), *F*(1, 1282) = 4.65, *p* < 0.05. The number of object count per an image segment was entered as a dependent variable in ANOVA by country (the U.S. and Japan), which shows the U.S. picture books (*M* = 20.07, *SD* = 29.44) contained significantly less number of objects than Japanese picture books (*M* = 26.64, *SD* = 47.08), *F*(1, 1127) = 9.71, *p* < 0.01.

**Figure 2:**
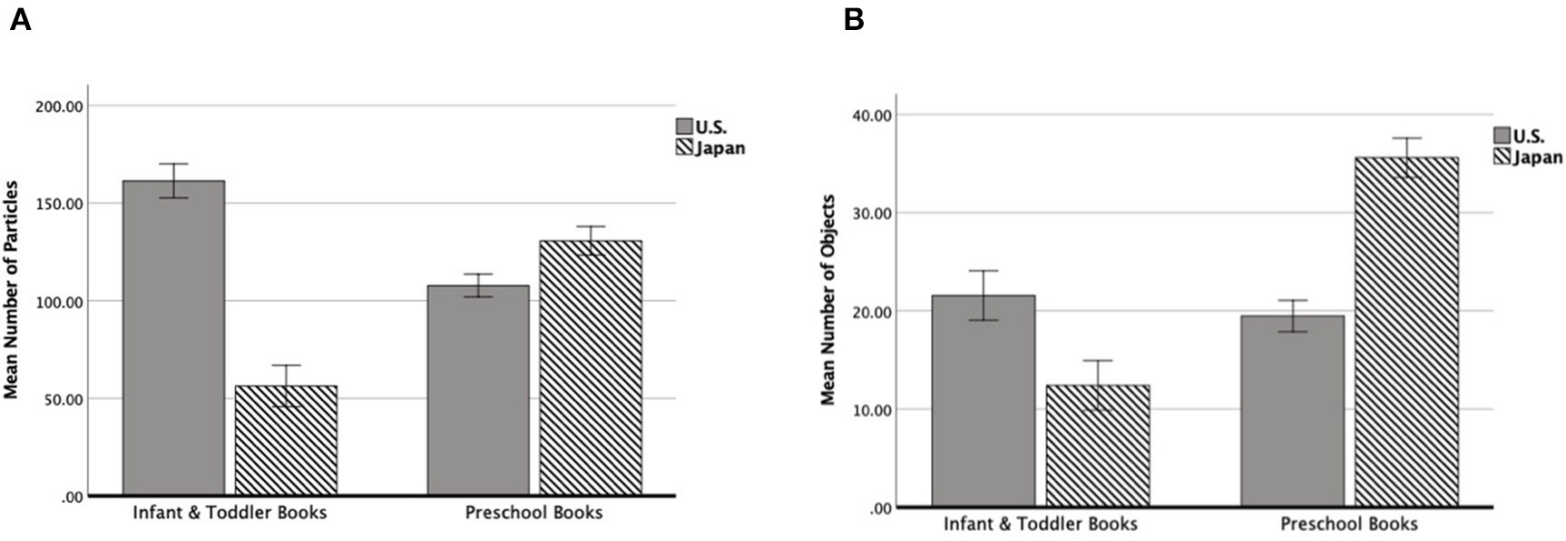
The bar graph of the average number of particles counted by the ImageJ **(A)** and the average number of objects counted by the human coders **(B)**. The x-axis shows the books targeting different age groups (infant/toddler and preschool) and the y-axis shows the mean number counted by each coding method. The gray bars are for the U.S. picture books and the striped bars are for Japanese picture books. The error bars were set as +/– 1 SE.

To see whether the target age of picture books influence the visual crowdedness, particle counts per an image segment was entered as a dependent variable in 2 (country—the U.S. and Japan) × 2 (age—infant/toddler books and preschool books) ANOVA. This yielded significant interaction between country × age, *F*(1, 1280) = 59.13, *p* < 0.01 (see Figure 2A). For infant and toddler books, the U.S. books (*M* = 161.32, *SD* = 139.93) contained significantly more particles than Japanese books (*M* = 56.29, *SD* = 72.47), *F*(1, 395) = 76.28, *p* < 0.01. For preschool books, the U.S. books (*M* = 107.78, *SD* = 136.41) contained significantly fewer particles than Japanese books (*M* = 130.67, *SD* = 150.19), *F*(1, 885) = 5.48, *p* < 0.05. Infant and toddler books (*M* = 161.32, *SD* = 139.93) contained significantly more particles than preschool books (*M* = 107.78, *SD* = 136.41) in the U.S., *F*(1, 778) = 25.03, *p* < 0.01, whereas infant and toddler books (*M* = 56.29, *SD* = 72.47) contained significantly fewer particles than preschool books (*M* = 130.67, *SD* = 150.19) in Japan, *F*(1, 502) = 35.38, *p* < 0.01. The number of object counts per an image segment was also entered as a dependent variable in 2 (country—the U.S. and Japan) × 2 (age—infant/toddler books and preschool books) ANOVA, which yield significant interaction between country x age, *F*(1, 1125) = 33.21, *p* < 0.01 (see Figure 2B). For infant and toddler books, the U.S. books (*M* = 21.56, *SD* = 33.10) contained significantly more objects than Japanese books (*M* = 12.40, *SD* = 25.13), *F*(1, 336) = 10.63, *p* < 0.01. For preschool books, the U.S. books (*M* = 19.47, *SD* = 27.83) contained significantly less number of objects than Japanese books (*M* = 35.58, *SD* = 54.85), *F*(1, 789) = 33.44, *p* < 0.01. Infant and toddler books (*M* = 21.56, *SD* = 33.10) and preschool books (*M* = 19.47, *SD* = 27.83) did not differ in the number of objects in the U.S., *F*(1, 762) = 0.79, *p* > 0.05, whereas infant and toddler books (*M* = 12.40, *SD* = 25.13) contained significantly less number of objects than preschool books (*M* = 35.58, *SD* = 54.85) in Japan, *F*(1, 563) = 34.38, *p* < 0.01.

To see whether the visual crowdedness coded by the ImageJ and the number of objects counted by human coders have any relationship, the mean number of particles was correlated with the mean number of objects, which yield significant correlation, *r* = 0.42, *p* < 0.01.

## Discussion

This study investigated the visual environment of young children to see whether there are cultural differences in what young children were exposed to. Specifically, we compared one of such visual environments, picture books in the U.S. and Japan. Based on previous studies, we predicted Japanese picture books would be visually more crowded than the U.S. books. Two coding methods we used in this study as a measure of visual crowdedness, the number of particles counted by ImageJ and the number of objects counted by the human coders correlated significantly, suggesting these two measures used in previous studies are related and both of them combined might be a good indicator of visual crowdedness. Although these two measures are related, the results show the different trend between the number of particles and the number of objects. As we expected, we found the U.S. books were less visually crowded than the Japanese books according to the number of objects coded by the human coders. However, the U.S. books were more visually crowded by including more particles than Japanese books by the ImageJ. This difference might be due to how we process holistic/global structure and analytic/local structure of scenes. Although we used two codings of the visual crowdedness based on previous studies, based on the previous studies of the visual development have shown human process the holistic/global structure (e.g., t-shirt) earlier and more predominant than the analytic/local structure of the visual scene (e.g., each line of the t-shirt), suggesting the number of objects might be a better measure than the particle measures (e.g., Kimchi, 1992; Johnson, 2010). This difference might also be due to the potential differences in how objects are emphasized in each country. Previous studies found objects, especially namable objects, were considered an important part of development in young children in the U.S., but not so for Japanese children (e.g., Gopnik and Choi, 1990; Fernald and Morikawa, 1993). Therefore, having less namable objects in the books might make it easier for U.S. children to pay attention to objects which are important in the U.S. society, but not necessarily so for Japanese children. For example, when looking at the videos of human actions, 24-month-old infants from the U.S. looked longer at the objects whereas infants from China looked longer to the actions (Waxman et al., 2016), suggesting it would be possible that the visual environment influences on cognition might start with objects.

For the number of objects, the results also showed no significant difference in the number of objects between infant and toddler books and preschool books in the U.S. This result might be due to that the U.S. books might not have clearer target ages as seen by libraries did not agree on the target ages of books whereas Japanese libraries agreed. If this was the case, the comparison should be all the U.S. books (because the target ages are not clear) and preschool books in Japan. With this analysis, we found U.S. books (*M* = 20.07, *SD* = 29.44) still contained significantly less number of objects than Japanese preschool books (*M* = 35.58, *SD* = 54.85), *F*(1, 1109) = 37.38, *p* < 0.01.

The results show the visual crowdedness of picture books varied based on the target age of the books. For picture books targeting infants and toddlers, the U.S. books contained a greater number of particles and a greater number of objects than Japanese picture books, which made the U.S. books more visually crowded than Japanese books. This is somewhat surprising given our expectations that Japanese books would be more visually crowded than the U.S. books. As expected, Japanese preschool books contained more particles and more objects than U.S. books, which made Japanese preschool books more visually crowded than U.S. books. The results suggest by preschool age, the visual contents of the picture books follow a very similar trend with other visual environments studied previously—the U.S. environment being simpler than the Japanese environment. The results also pose the possibility that the change in the visual crowdedness—the decrease in the visual crowdedness in the U.S. books from infant and toddler books to preschool books and the increase in the visual crowdedness in Japanese books from infant and toddler books to preschool books—might provide needed opportunities for comparison, which has a central role in other cognitive domains, such as categorization, memory, and similarity judgments (e.g., Gentner and Medina, 1998). Therefore, the change in visual crowdedness, which provides a comparison process, might be necessary for attention and perception to be attuned to visual environment differences.

Although we have created a new database of books to be used for cultural comparison in future studies, the number of books included in this study based on the inclusion criterion that is not culturally biased was small that might need to be expanded in the future. Also, besides the cultural differences in visual contents of picture books which we found, the visual environments that young children encounter are not limited to picture books. Studying other visual environments that young children are regularly exposed to would be a vital next step to further our understanding of these visual environments influences on cognition. Despite these limitations, the current finding reflects the similar trends found in the current literature on attention and perception, indicating visual environments that young children are exposed to regularly might influence the cultural differences in attention even in young children. Previous studies have found the direct link between visual environment and people's behaviors (e.g., Miyamoto et al., 2006; Tsai et al., 2007). For example, Tsai et al. (2007) found priming children with an exciting story made those children choose the exciting activity over calm activity. Therefore, future studies could use picture books as primes to see whether the visual contents of picture books could act as a direct influence on cultural differences in attention. The finding from infants and toddler books also provides the potential new line of research, suggesting young infant and toddlers might not show the expected cultural differences in attention based solely on picture books, but needs multiple visual environments differences to influence their attentional systems. Because the book reading is a shared activity between adult and child, it would also be interesting to see how adults read books to children might differ based on the visual contents of books (e.g., one with fewer objects vs. one with more objects). Because previous research has shown picture books help children's development, such as language (e.g., Bus et al., 1995), socio-emotional understanding (e.g., Adrian et al., 2005), and memory (e.g., Cornell et al., 1988), how the differences in visual content might influence these development domains would be other future interests. Further, because the picture books contain both the images and wording, it would be interesting to see how the visual properties might be related to the wording of the image.

We understand the environment (e.g., physical, social, visual) and our mind interacts and constructs cultural differences (Shweder et al., 2006). For example, the ways humans create these cultural products might be influenced by the minds of creators, which in return influences other minds. However, as pointed out by Morling and Lamoreaux (2008), the research fields have focused on the differences in our minds over the environmental differences. Therefore, the systematic analysis of the environment would give us insight into how our minds interact with the environment differently. In this study, we explored the potential cultural differences in such an environment for young children as a first step into untangling the interaction between culture and our minds.

## Data Availability Statement

The datasets generated for this study are available on request to the corresponding author.

## Author Contributions

MK contributed to all aspects of this research, the conception of the idea, designing, data coding, analysis, and drafting the article. JA and DA contributed to the conception of the idea, designing, data coding, and drafting the article.

### Conflict of Interest

The authors declare that the research was conducted in the absence of any commercial or financial relationships that could be construed as a potential conflict of interest.
