# Supplementary material for: Cultural Differences in Visual Contents in Picture Books
Source: Front Psychol. 2020 Feb 25;11:304. doi: 10.3389/fpsyg.2020.00304 (PMC7052262; doi:10.3389/fpsyg.2020.00304)
Supplement: Supplementary file 1 [file Table_1.pdf]

Appendix A

List of picture books (title, author name, and illustrator name) included in this study by country and target age

The U.S. picture books for preschoolers

Title	Author	Illustrator
Bark, Geroge	Jules Feiffer	Jules Feiffer
Brown Bear, Brown Brear What Do You See?	Bill Martin, Jr.	Eric Carle
Caps for Sale	Esphyr Slobodkina	Esphyr Slobodkina
Chicka Chicka Boom Boom	Bill Martin, Jr., and John Archambault	Lois Ehlert
Corduroy	Don Freeman	Don Freeman
Curious George	H.A. Rey	H.A. Rey
Don't Let the Pigeon Drive the Bus!	Mo Willems	Mo Willems
Duck on a Bike	David Shannon	David Shannon
Eating the Alphabet	Lois Ehlert	Lois Ehlert
Gossie	Oliver Dunrea	Oliver Dunrea
Harold and the Purple Crayon	Crockett Johnson	Crockett Johnson
Harry, the Dirty Dog	Gene Zion	Margaret Bloy Graham
If You Give a Mouse a Cookie	Laura Numeroff	Felicia Bond
Make Way for Ducklings	Robert McCloskey	Robert McCloskey
Officer Buckle and Gloria	Peggy Rathmann	Peggy Rathmann
Olivia	Ian Falconer	Ian Falconer
Pete's a Pizza	William Steig	William Steig
The Napping House	Audrey Wood	Don Wood
The Snowy Day	Ezra Jack Keats	Ezra Jack Keats
The Story of Ferdinand	Munro Leaf	Robert Lawson
Trashy Town	Andrea Zimmerman & David Clemesha	Dan Yaccarino
Tuesday	David Wiesner	David Wiesner
Where the Wild Things Are	Maurice Sendak	Maurice Sendak

The U.S. picture books for infants and toddlers

Title	Author	Illustrator
**Big Fat Hen**	**Keith Baker**	**Keith Baker**
**Freight Train**	**Donald Crews**	**Donald Crews**
**From Head to Toes**	**Eric Carle**	**Eric Carle**
**Goodnight Moon**	**Margaret Wise Brown**	**Clement Hurd**
**I Kissed the Baby**	**Mary Murphy**	**Mary Murphy**
**Knuffle Bunny: A Cautionary Tale**	**Mo Willems**	**Mo Willems**
**Mama Cat Has three kittens**	**Denise Fleming**	**Denise Fleming**
**My Car**	**Byron Barton**	**Byron Barton**
**My Very First Mother Goose**		**Rosemary Wells**
**Peekaboo Morning**	**Rachel Isadora**	**Rachel Isadora**
**Ten, Nine, Eight**	**Molly Bang**	**Molly Bang**
**The Very Hungry Caterpillar**	**Eric Carle**	**Eric Carle**
**Where's Spot?**	**Eric Hill**	**Eric Hill**

The Japanese picture books for preschoolers

Title	Author	Illustrator
11ひ ゚きのねこ	馬場のほ ゙る	馬場のほ ゙る
14ひきのひ ゚くにっく	いわむらかず ゙お	いわむらかず ゙お
おしゃへ ゙りなたまこ ゙やき	寺村輝夫	長新太
おとうさんあそぼう	わたなべしげお	おおともやすお
おふろた ゙いすき	松岡享子	林明子
かさじぞう	瀬田貞二	赤羽末吉
かちかちやま	おざわとしお	赤羽末吉
かばくん	岸田衿子	中谷千代子
からすのハ ゚ンやさん	かこさとし	かこさとし
きょだいなきょだいな	長谷川摂子	降矢なな
ぐりとぐら	中川李枝子	大村百合子
ぐるんぱのようちえん	西内ミナミ	堀内誠一
しょうぼうじどうしゃじぷた	渡辺茂男	山本忠敬
しろくまちゃんのほっとけーき	わかやまけん	わかやまけん
ぞうくんのさんぽ	なかのひろたか	なかのまさたか
だいくとおにろく	松井直	赤羽末吉
ねず ゙みくんのチョッキ	なかえよしを	上野紀子
はし ゙めてのおつかい	筒井頼子	林明子
ほ ゙くのくれよん	長新太	長新太
みんなうんち	五味太郎	五味太郎
めっきらもっきらと ゙おんと ゙ん	長谷川摂子	ふりやなな
やさいのおなか	きうちかつ	きうちかつ
わたしのワンピース	西巻芽子	西巻芽子

The Japanese picture books for infants and toddlers

Title	Author	Illustrator
あがりめさがりめ		ましませつこ
いない　いない　ばあ	松谷みよ子	瀬川　康男
おつきさま　こんばんは	林明子	林明子
かおかおと゛んなかお	柳原良平	柳原良平
がたん　ごとん　ガタン　ごとん	安西水丸	安西水丸
きんき゛ょか゛にけ゛た	五味太郎	五味太郎
くだもの	平山和子	平山和子
くっついた	三浦太郎	三浦太郎
じゃあ　じゃあ　びりびり	まついのりこ	まついのりこ
たまごのあかちゃん	かんざわとしこ	やぎゅうげんいちろう
ねないこた゛れた゛	せなけいこ	せなけいこ
ひ゜ょーん	まつおかたつひて゛	まつおかたつひて゛
もう　おきるかな？	まつのまさこ	やぶうちまさゆき
もこ　もこもこ	谷川俊太郎	元永定正
